# Resilience in Times of Economic Boom and Bust: A Narrative Study of a Rural Population Dependent upon the Oil and Gas Industry

**DOI:** 10.1007/s10804-020-09363-z

**Published:** 2020-10-22

**Authors:** Hamideh Mahdiani, Jan Höltge, Linda Theron, Michael Ungar

**Affiliations:** 1 Institute for History, Theory and Ethics of Medicine, University of Mainz, Am Pulverturm 13, 55131 Mainz, Germany; 2 Resilience Research Centre, Dalhousie University, Halifax, NS Canada; 3 Department of Educational Psychology, Faculty of Education, University of Pretoria, Pretoria, South Africa

**Keywords:** Adult resilience, Boom and bust economic cycles, Narrative analysis, Oil and gas industry, Life-story construct

## Abstract

How do residents of small towns that depend on oil and gas extraction or processing industries withstand economic boom and bust cycles? To answer this question, this article reports on a narrative analysis of residents’ life stories gathered from 37 adults of a small town on the Canadian prairies dependent on the oil and gas industry, employing the theories of narrative inquiry and narrative identity. Participants aged 30 to 76 were interviewed and their experiences of living in an unstable economy that is dependent mostly on a single resource extraction industry were explored. Specifically, we asked participants about the effect of economic change on factors related to resilience like family interactions, work choices, educational pathways, and the quality of their social lives. Our analysis of adult narratives looked for patterns in the relationship between risk exposure, promotive and protective factors at multiple systemic levels (individual, relational, cultural), and functional outcomes such as individual coping, community cohesion, and social and economic sustainability. Results show that a strong identity, in particular expressions of personal agency, communion, and engagement in meaning making are contributing factors to adult resilience in a context of economic change. Our results also highlight how positive attitudes towards a better future may inadvertently undermine the need for residents of oil and gas-dependent towns to commit to economic diversification and other potential resilience-promoting strategies.

**Electronic supplementary material:**

The online version of this article (10.1007/s10804-020-09363-z) contains supplementary material, which is available to authorized users.

## Introduction

Psychological resilience in adulthood is defined as individuals’ ability to “maintain relatively stable, healthy levels of psychological functioning” when facing “highly disruptive events” (Bonanno 2004, pp. 20–21). Based on different studies by Bonanno and colleagues (Bonanno et al. 2002, 2005, 2006) it is maintained that individuals who are exposed to atypical stressors generally show psychological resilience, and despite temporary setbacks, maintain their normal level of functioning. One such disruptive event is an unstable economy and the problems caused by commodity price fluctuations for people living in resource-dependent communities.

All economies, local, community, regional, and national, are vulnerable to boom and bust cycles characterized by phases of expansion, peak, contraction, and trough. Whereas psychological resilience is dependent on an individual’s ability to make use of available social–ecological resources (Ungar and Theron 2020; Masten and Motti-Stefanidi 2020)*,* economic resilience is defined as “the policy-induced ability of an economy to withstand or recover from the effects of […] shocks” (Briguglio et al. 2006, p. 265) and the ability to cope with shocks through counteractive mechanisms. There is, however, a tension between psychological and economic approaches to resilience with policies that support economic resilience dependent, in part, on individual psychological resilience for their actualizations. Individual, community, and economic resilience seem to be not only interrelated, but also context-dependent (Ungar 2012). To illustrate, Lapuh (2018), through the study of socio-economic structure in Slovenian municipalities in 2008, concluded that the focus of “territorial policy” should be directed towards “entrepreneurship and ensure a diverse business structure” (p. 154) if the goal is to be more resilient. Such findings testify to the need to account for the actions of individuals (e.g., entrepreneurs) when considering broader economic processes. Therefore, interventions that bolster the psychological resilience of individuals (e.g., decreasing unemployment or supporting agency) will exert a positive influence on multiple systems at the same time (individual mental health, workplace productivity, national GDP, etc.) (Powdthavee 2012; Winklemann and Winkelmann 1998).

It follows, then, that communities that depend on a single resource extraction or processing industry, such as oil and gas, may be particularly vulnerable as shifting commodity prices and changing social behaviors (e.g., movement towards a low-carbon or zero-carbon economy) put additional pressure on community residents to adapt. Although research on economic resilience has measured different factors of socio-economic resilience regarding regional resilience (Christopherson et al. 2010), urban resilience (Leichenko 2011; Tyler and Moench 2012), or a general adaptation to environmental change (Nelson et al. 2007) among others, in all such examples the emphasis has been given to “a resilient region, in environmental and economic terms, one that retains the capacity to recover from external shocks” (Christopherson et al. 2010, p. 5). We believe that a comprehensive study of resilience has the potential to shift the focus from either regional economic resilience or psychological resilience and instead show the inter-dependence of resilience-promoting processes across social–ecological systems (Ungar 2012). Such a focus disputes an individualistic and reductionistic approach to resilience and instead focuses on the contextualized reactions of individuals in relation to the changing availability of resources (Ungar 2012).

In order to study such interactions, we look at individual narratives of resilience in the context of economic stress. Though our population is unique, a few previous studies have also accounted for economic conditions and individual resilience narratives. For example, Canvin et al. (2009) focused on narratives collected from interviews drawn from the youth and adult sample of the British Household Panel Survey to study resilience processes among households experiencing material hardship in Britain. They concluded that adults’ psychological responses to economic challenges are partially dependent on distal childhood experiences rather than more proximal factors like personal finances, employment status, or training. Of relevance to our project is Canvin et al.’s emphasis on asking *people* living in poverty what *they* see as achievements and positive transitions in their lives and identifying the resources (e.g., self-esteem and social support) they have depended on over time to cope with economic conditions. Research on the topic in the American context also supports similar conclusions (see Ennis 2015; Gourley and Madonia 2018; Haggetry 2014; Pippert 2018). Moreover, longitudinal studies have, in recent years, shown how quickly residents of an economically challenged environment can adapt in ways that maintain mental health (Frijters et al. 2011).

Our study focuses on a small community on the Canadian prairie, Maple Hill^1^, that experienced growth over the past 50 years following expansion of the oil and gas industry (current population of the town and the surrounding county is 14,600). During the 1970s, with oil prices soaring, rural areas in regions like the province of Alberta saw their populations increase by a third followed by a construction boom that outpaced larger cities on a per capita basis (*Boom and Bust in Alberta* 2001). Since then, the economy has been through ups and down, with a major decline starting in 2014 when oil prices dropped.

Given the lack of studies which account for the residents’ perspectives on resilience to boom and bust cycles, our study is a narrative enquiry of what the resident adult population have to say about their boom and bust experiences within individual, communal, and social contexts. Therefore, in order to answer our main question which is what capacities the adults of this small town have to deal with the multiple challenges they face, our interview questions targeted their individual, social, and work life. The participants’ answers were explored for potential promotive and protective processes of resilience with the goal to provide a new understanding of resilience in economically adverse contexts which benefits from the residents’ narratives and narrative identity as organizing concepts for the data. In the next section we elaborate on these two terms.

## Narrative, Narrative Identity, and Resilience

At its most simplistic, a narrative is a story with a beginning, a middle and an end (Atkinson and Delamont 2008). The recent interest in the study of narratives beyond the fictional/semi-fictional and literary domain has initiated the *narrative turn* not only in the humanities but within fields as diverse as sociology, medical science, and law. By extension, narrative analysis can be viewed as an approach towards understanding humans’ perception of life and its meaning.

The number of studies exploring the relationship between narratives and resilience is growing with contributions from fields as diverse as gerontology (Randall et al. 2015) and talk therapy (White 2007). In general, using narratives as both a “conceptual framework and a research methodology” (Clark et al. 2018, p. 65) can serve the purpose of not only studying resilience (whether it is a process or an outcome) but can also “provide important insight into the lived experience of stressors and resilience.” Other researchers have gone a step further and created what has been termed a “grammar of resilience” through the study of narratives (Ramsey and Blieszner 2013). Ramsey and Blieszner (2013), for example, found that factors such as how much detail an adult’s story has, what their narrative tone is, which genre was the most used, whether the adult is the author, hero, or the villain of their story, or the metaphors, motifs, and themes (in Randall et al. 2015) are all narrative components relevant to understanding coping under stress. It is also assumed that “adults who score high on resilience will story their lives in identifiable ways” (Kenyon et al. 2010), as identified in Ramsey and Blieszner’s grammar.

For the purposes of this study, we build on evidence that the presence or absence of specific themes in an individual’s life story can reflect resilience or lack thereof. The themes are based on McAdams’ theories of narrative identity (2013). McAdams provides a comprehensive categorization of *life-story constructs* which are employed in studies of narrative identity (McAdams and McLean 2013). McAdams (1985) formulated a life-story model of identity, contending that people living in modern societies begin, in late adolescence and young adulthood, to construct their lives as evolving stories. He defines narrative identity as “a person’s internalized and evolving life story, integrating the reconstructed past and imagined future to provide life with some degree of unity and purpose” (McAdams and McLean 2013, p. 233). Similarly, Pals’ (2006) work highlights the relation between *explorative narrative processing* and how people make sense of difficult life experiences. Pals writes that those adults who face adversities in their adult life may have more trouble constructing their identity. In other words, it is through narrative identity that individuals communicate their being in time, past, present, and future. Following this conceptualization of identity as storied, McAdams and McLean have identified a coding structure based on seven life narrative constructs: *agency, communion, meaning making, explorative narrative processing, coherent positive resolution, redemption,* and *contamination.* We see links between McAdams and McLeans’ life-story constructs of narrative identity and the core concepts of social–ecological (Ungar 2012) or systemic (Masten 2014) resilience as found in resilience researchers’ understanding of resilience and identity development in stressed environments. Each of these associations is exemplified below:

*Agency* refers to the extent an individual can take action to develop mastery over their life or others’ lives (McAdams and McLean 2013). McAdams and McLean (2013) explain adults with life stories which reflect the theme of agency under stressful situations tend to have higher levels of mental health, well-being, and maturity. Similarly, Ungar et al. (2007) identified seven critical factors associated with resilience across different cultures which are also related to adaptation and meaning making, namely: relationships, powerful identities, experiences of efficacy, social justice, the meeting of basic needs, social cohesion, and cultural adherence. The two factors of *powerful identities* and *experiences of efficacy* are synonymous with the role of agentic narratives. Ungar (2015) defines powerful identity as a strong personal and collective sense of purpose, or self-appraisal of strengths and weaknesses. He further related self-efficacy to the ability to affect change in one’s social and physical environment in order to access health resources. Ungar’s research, like McAdams and McLean’s, associate resilience with highly agentic individuals in environments that facilitate personal growth and development.

*Communion* is defined as the extent individuals portray communal connections (McAdams and McLean 2013). Stories rich in communion emphasize “intimacy, caring, and belongingness” (McAdams and McLean 2013, p. 234). Similarly, social–ecological accounts of resilience underscore the role of relationships with significant others, including peers and adults within one’s family and community. For example, Crummy (2002) studied the effect of relationships as a key resilience factor for bereaved older adults. Luthar and Brown (2007) claimed that resilience hinges on relationships. Accordingly, relationships which are rooted in abusive behaviors jeopardize resilience, whereas “love, comfort and security foster resilience” (Panter-Brick and Eggerman 2012, p. 370) and reflect the role communion plays in well-being under stress.

*Meaning making *as a life narrative construct is defined as the extent of learning from an adverse experience (McAdams and McLean 2013). In a longitudinal study investigating whether recognition of meaning in life is related to resilience to suicide, Heisel and Flett (2016) identified perceived meaning in life as an important factor, as did Edward et al. (2009) whose work focused on how adult patients of mental health services who have experienced mental illness describe the phenomenon of resilience. Theron and Theron (2014), also studied meaning making processes among black South African students in a broader pursuit of resilience-promoting processes. In all these examples, a meaningful relationship between meaning making and individual resilience has been observed.

*Exploratory narrative processing* is the degree of “self-exploration as expressed in the story” where a resilient individual’s narrative would reflect “the development of a richly elaborated self-understanding” (McAdams and McLean 2013, p. 234). An exemplary study in the field of resilience research is the work by Lau and van Niekerk (2011) on narratives of resilience among burn survivors in which the researchers reflect on the role of exploratory narrative processing. Lau and van Niekerk engaged with their participants through their stories where the participants were asked to reflect on their traumatic experiences. They found that individuals’ narratives were embedded with themes of “growth and transformation” as their stories demonstrated how they learned positive lessons from traumatic childhood experiences. Their findings also reflect on the role of narratives and meaning making processes in relation to the survivors’ “reconstruction of self,” where the dominant positive narratives reflected “self-enhancement, increased clarity of identity and life purpose, insight into self, and meaningful connections with others” (p. 1175).

*Coherent positive resolution* is how much closure is produced in a story (McAdams and McLean 2013). For example, studies by King and Hicks (2007) and Lilgendahl and McAdams (2011) prove how positive resolution of negative events is associated with higher levels of happiness and well-being. Similarly, resilience research underscores this factor. For example, Pals’ research (2006) on exploratory narrative processing, although dedicated to the field of narrative analysis, concluded that coherent positive resolution also “predicted increasing ego-resiliency between young adulthood and midlife (age 52),” which itself “mediated the relationship between coherent positive resolution and life satisfaction in late midlife” (1079). Similarly, Compton and Hoffman (2019) related human resilience to coherent positive resolution and happiness. Also of particular importance is Fredrickson’s *broaden and build* theory of positive emotions (2001). She posits that positive emotions, such as “joy, interest, contentment, pride, and love” (p. 3), can provide people with lasting resources. Elsewhere, she measured psychological resilience of participants during a speech task, and concluded that more resilient individuals reported greater happiness.

*Redemption* refers to the storylines where there is a positive outcome rooted in a negative experience (McAdams and McLean 2013). The initial negative state is compensated for by the good that follows it. Returning to Lau and van Niekerk’s (2011) research, participants’ stories which demonstrated their transformation and growth out of traumatic childhood experiences mirror such a theme. Furthermore, resilience scholars underscore the role of reappraisal (Kalisch et al. 2015) and optimism (Rutter and Pickles 2016), concepts reminiscent of the theme of redemption discussed by McAdams et al. (2013).

*Contamination *is the opposite of redemption where a negative outcome is reached based on a positive experience (McAdams and McLean 2013). This factor, by its definition, should not be a frequent construct in resilience-themed narratives.

In summary, we used all of McAdams’ constructs to inform a structured analysis of the narratives collected from the residents of the town of Maple Hill. These narratives detail residents’ accounts of lives lived during boom and bust economic cycles in a socioeconomically vulnerable context. Using McAdams’ constructs facilitated attention to resilience in this context.

## Methodology

Our methodology uses Polkinghorne’s theories of narrative inquiry (1995) to analyze the adult participants’ life narratives. This provides an adequate tool to study McAdams’ life narrative constructs in relation to the resilience of adults who have lived through boom and bust cycles. Polkinghorne has introduced two versions of narrative inquiry^2^: *analysis of narratives* (identification of common themes across a series of narratives), and *narrative analysis* (taking an in-depth look at one narrative to identify the aspects of narrative processes, cognition, tools, and language used). In *analysis of narratives*, based on a comparative look into the totality of the stories told, we create a new structure. This requires the collection of stories as data, followed by paradigmatic analysis that results in “descriptions of themes that hold across the stories” (Polkinghorne 1995, p.12). For the purpose of this paper, we are interested in those themes relevant to McAdams’ life-story construct which illustrate the presence, absence, or degree of adult resilience. In contrast, *narrative analysis* is concerned with perception through the plotline of an individual story. The result of narrative analysis is itself a story (Smith and Sparkes 2009).

What is important from a narrative perspective is the way in which events and experiences are connected and the process through which meanings are produced, rather than whether the events are true in themselves (Trapp-Fallon 2003). This method has been used by narrative analysts including McCance et al. (2001) who identified common themes using paradigmatic analysis of narratives to construct six stories of nurses’ experiences of delivering care. Likewise, Cussen et al. (2012) used narrative analysis to construct a set of common themes that explain adolescents’ aspirations for the future. Other researchers have employed Polkinghorne’s dual techniques successfully (see Kramp 2004; McCormack 2004).

To sum up, on the one hand, our analysis of narratives seeks to produce a unifying narrative which expresses the totality of the stories heard from the participants. In the section on *narrative analysis*, on the other hand, we compare the recurrent themes deduced from the narratives with those of McAdams detailed earlier.

### Sample

We interviewed 37 adults (20 women and 17 men) between the ages of 30 and 76 (mean age = 51.51 years) who had resided in Maple Hill for an average of 27.5 years, with the minimum of 2^3^ and maximum of 63 years. The participants were recruited either by responding to the social media platforms of the project and town or in person through contact with onsite researchers, for example during community dinners, or weekend farmers’ markets.

In our sample, only one participant had indigenous roots, and one was in a same-sex relationship. The majority of the participating adults represented heterosexual partners with children.

### Study Design and Procedure

Following Glaser and Strauss’s iterative-cyclical process (1967), we conducted three rounds of interviews:

In the first round, six interviews were completed using open questions related to the topic of resilience. The data collected from this round were then analyzed and key themes such as family and community factors were identified. These themes were used in the second round, 14 interviews, with specified questions on resilience in boom and bust cycles. In the last round of interviews, with 17 participants, we included questions about private lives, work lives, community lives, and future lives in the context of oil and gas industry cycles. For example, “How does the oil and gas industry affect your family life in relation to your partner, children and the family as a whole?” “What ways have you and your family found to handle the challenges of the boom and bust industry?” and “How does the oil and gas industry affect your social network in Maple Hill?” (see “*Appendix A*” for the final interview guide). Participants were not asked directly to define resilience (only two participants used the term in their responses). During all the interviews two researchers were present who specialized in resilience research with sub-specialities in social studies, psychology, and narratology.

All interviews were digitally recorded (with permission of the participants) and coded using ATLAS.ti 8 (2017), a software for the storage, management, analysis, and retrieval of qualitative data. When coding the data, the analytical strategy and focus were discussed by all the authors and a consensus reached regarding the grouping of the codes. Data used for this article were grouped under narrative identity and focused on identifying McAdams’ seven constructs. The focus of this article is only one part of the analysis conducted on the collected data. Coauthors have produced different analytical reports and articles, ranging from a gender-specific reading (Murphy et al. (under review)) to the role of industry (Twum-antwi et al. (in press)), all under the larger frame of resilience research.

Before every interview, the participants were informed of the details and purpose of the study and the confidentiality of their data. All the interviews took place at the same office building provided to the research team by the town’s municipal government. Each participant was interviewed only once. The interviews were planned to last 1 h and organized to have more time if needed. While the main topics of the guide were broadly covered in each interview, the interviewers were flexible in focusing more on specific topics in relation to the participants’ story. Due to the relatively small size of the community, the participants might have known about one another’s participation in the study though this appears to have had no effect on the data or our analysis based on the feedback from participants and the local advisory committee which oversaw the administration of the research.

## Findings

### Narrative Analysis: The Complexity of Boom-Bust Adjustment

Complexity in the narratives was most evident when we examined people’s accounts of their lives during both boom and bust periods. Contrary to expectations and given the preference people tend to have for economic booms, participants talked more about the *challenges* boom times brought to individuals and families rather than the advantages. During boom cycles, there were jobs and good money, but that also meant that fathers/male spouses were away at work for periods of time that lasted days, weeks, or months (all our examples were of men being removed from the family, even though an increasing number of women are now working in the oil and gas industry or in male-dominated professions). Most of the female participants experienced periods as single parents or had to take on extra responsibilities for the household or a farm. This occurred most often during economic booms. For example, one female participant said: “My husband, he works far away though so it’s difficult and he’s also in his own very stressful situation” (Female, age 34). Another said, “It took a while for that adjustment […] so it was hard being at home all the time and you’re with somebody and they’re always gone. So that was hard to adjust” (Female, age 49). For her, ‘adjustment’ came with, on the one hand, being trained as a school bus driver and not only driving her own children everyday, but meeting many parents and expanding her social circle, communicating and socializing on a daily basis. The protective role of social networks is a proven resilience factor, referred to by Richardson (2002) as ‘social reintegration’ (see also Wells 2012). She also talked about her own skills as a single mother before remarrying in “budgeting and managing.” On multiple occasions she underscores how learning to save money and budgeting was a key adjustment factor. In resilience studies, characteristics like self-organization and learning new knowledge have been observed as mediating resilience. The role of learning to adjust was recounted by other participants as well. For example, when men returned from work, the pressure to adapt to stressful schedules did not end. Participants described periods of adjustment following long absences:

When he is home and you do make decisions, you communicate, but it takes a lot to work like, you have to be strong about it […] he was so used to just doing everything his way and I was doing everything mine and then he’d come home and he’d try to change things and I’d be like, ‘Yeah, no’ and so it took a while. (Female, age 49).

Likewise, some participants said that their family acts differently during the periods of time that the father is home; “We have a system in place, when he’s not there and then he comes home” (Female, age 49). However, descriptions of learning and adjustment did not come from women only. In some cases, it was men who talked about the difficulties their jobs caused them in relation to their families. For example, one father said, “I wish I had more time with my son, him being the oldest, could have done things” (Male, age 72). Another man talked about how shift work during boom periods made him emotionally distant: “I think I was a zombie. Yeah. If I was talking to you, I wouldn’t even remember, sometimes, it got pretty bad” (Male, age 72).

Whether it was because of the financial incentives to work long hours, or the necessity to maintain their family, participants described learning to adjust to the demands caused by boom time conditions, on in other words. As one female participant explained: “He’d work out of town a lot and I structured my life so that I could go see him” (Female, age 50). To her, the advantage of her husband’s work was a paycheque “10 times” larger than her salary which made the sacrifices worthwhile. Another participant described the economic advantages of stressful work life as a blessing that demanded compromise: “I feel so blessed. Like, we put our family first. If it meant us going and staying in a hotel with them for a weekend, then we did that because the family unit was really important […] wherever he needed to go, we went” (Female, age 50). In each example, participants described ways they had maintained a sense of cohesion among family members even if the transitions from work to home life caused tension for both spouses and children when work was plentiful and the financial benefits very good. In short, boom cycles stressed residents’ personal lives, but they learned how to adjust, be it by learning new skills (bus driver) and creating broader social circles, or rescheduling family life around the husband or father’s work shift, or simply weighing the benefits of a substantial income over less family time. In each case, the change in their approach of handling the situation did not occur overnight, rather they have developed new coping styles over time.

While we expected bust times to bring narratives of loss and stress as jobs became fewer and the pay lower, the pattern of responses was much more complex. For example, one participant explained the ambivalence experienced during a period of economic contraction: “Dads are at home right now. So, that’s impacting the family in some ways positively but in a lot of ways negatively” (Female, age 50). Such changes disrupted family life, with children and spouses unaccustomed to seeing fathers at home so often. Despite the challenge, this participant said that she thinks during economic downturns, “Societies are putting on a lot more activities for people to be social and be together, for families to be together,” which also represents the developments taking place in the context of social support.

This same ambivalence regarding the advantages and disadvantages of an economic bust was evident in the way men and women described their roles and the need to challenge traditional gender norms in order for families to adapt successfully. A 46-year-old male participant, for example, talked about how he is staying home while his wife makes money as a minister: “I was able to stay home for the first year of my now-4-year-old’s life (…) it was great.” Although he is not the breadwinner of the family any longer, he gets to enjoy family life and raise his children which reflects degrees of *positive adaptation*. Positive adaptation is usually defined as “success at meeting stage-salient developmental tasks” (Luthar and Zigler 1991). This same pattern was found among other male participants. As one man living common-law with his partner explained:

I’m usually pretty adaptive to change. It has been three times over the last 10 years of this industry that I haven’t been able to work either injury or just the last downfall […] it’s not pleasant because I prefer to work, he prefers to stay home, but we understand that reality is never nice, so that’s what we do. (Male, age 36).

These participants, 46 and 36 years old, are both exemplars for adults’ developmental behavior against challenges, whereby after a period of time they not only learn how to adapt to the unwanted change but also start seeing it not as a merely negative modification.

This developmental change presented itself in other aspects of the participants’ lives as well. Though participants noted that communities pulled together during economic busts, the quality of those interactions changed. Many participants said they had stopped having “drink nights” (going out to a bar with friends) as often as they used to. They mostly attended community events instead, or made greater use of public facilities like libraries and recreational spaces that had low or zero fees. For example, one male participant in his mid-50s said, “I’m starting to attend my church more…becoming more spiritual.” This is not to say that participants did not spend money on alcohol. One participant explained: “I think the liquor stores are busier, none of them have closed down” (Male, age 51). Consistently, we found that when drinking alcohol was part of a participant’s narrative, there was a shift in what it meant depending on whether the economy was doing well or poorly. During bust times, casual drinking changed from an opportunity to socialize to a coping strategy used to cope with feelings of failure. As one participant said: “I think people reach out to different things [during a bust] they feel might be able to help them cope…churches and liquor stores” (Male, age 30). Both spirituality and alcohol consumption can initiate participation in communal activities, where the individual can seek comfort in the company of others.

With regard to participants’ work lives, economic busts changed how much people earned, but did not improve work-life balance. As one participant said: “They paid you well [during the boom] but there was a lot of what I call criminal exchange, like, we want you to work, you know, 12–14 h days, 7 days a week until you’re done, and yes you got paid” (Female, age 50). Though the experience may have had its advantages (e.g., more discretionary income), bust periods offered an opportunity to slow down and recuperate. As another participant said: “When there is a boom, you work till you drop […] because you never know when it’s not going to be there” (Female, age 56). Busts brought opportunities to establish balance, even if that balance was not explicitly valued.

Examined altogether, people’s experience of family, community, and work lives through both boom and bust economic cycles is complex with multiple and competing narratives that account for the impact of a good or bad economy on how well individuals, families, and communities function. While the substance of the stories men and women in Maple Hill told was the same, the experience of each economic period meant a different set of adaptations and stressors for each gender. We can group the participants’ experiences of both boom and bust economies into three interactive storylines:

1. *Positive storylines*: Participants adjusted to the prosperity or the economic downturns having learned that life in a town dependent on the oil and gas industry requires flexibility. Such stories benefited from protective factors such as learning new skills, budgeting, and social networks.
2. *Neutral storylines*: Participants accepted that conditions would always be stressful. These stories portrayed a narrative tone which was neither positive nor negative. Life for these people seemed to be always the same.
3. *Negative storylines*: Family and community life is negatively affected by both good and bad economic conditions, with both booms and busts causing spouses to be absent (physically and emotionally). Financial supports are either abundant or missing, with both conditions causing strained social relationships and contributing to substance abuse.

Regardless of which storyline was more dominant in the individual’s narrative, both boom and bust periods demanded a great deal of personal and collective resilience to cope with the stress caused by a changing economy. The importance of both personal and collective resources fits with social–ecological or systemic approaches to resilience that discourage accounts of resilience that report individual strengths without acknowledging that these strengths are invariably intertwined with the strengths of the human and structural systems that individuals are connected to (Ungar and Theron 2020; Masten and Motti-Stefanidi 2020). In our sample, adults’ developmental growth in facing new challenges reflects the role of personal and collective resources.

### Analysis of Narratives

In order to better understand the relationship between narrative identity and participants’ resilience, we invited the participants to tell us about their lives in the form of a story (in the last round of the interviews, *n* = 17). A review of the plotlines, narrative tone, and the degree of complexity in the participants’ responses became the basis for an analysis of narratives and a deeper understanding of resilience processes in this unique context. For our analysis, we return to McAdams and McLean’s (2013) life-story constructs. Each of the seven aspects of narrative construction and identity development were reflected in the complexity of the stories that participants shared.

#### Agency

All the participants had experienced boom and bust cycles, with many commenting on the level of control they exercised in their lives regardless of economic conditions. For example, some described themselves as very independent: “I have a very different perspective…I did my own thing” (Female, age 39). Another participant said, “I’m, I’m a very uh, dominating person like, you know, I just, like, I said, this is what I’m doing, and I did it” (Female, age 63). Yet another said, “I was a very independent person and I looked after my own stuff” (Female, age 49). These participants all demonstrated a high agentic character reflecting empowerment and self-mastery.

Other participants maintained a more flexible attitude towards the changing economic conditions. For example, one said that “There’s no hard feelings here or anything else, whatever. Any place you make it work… all depends on your attitude” (Male, age 60). This was similar to another response, “It didn’t affect me that way kind of thing, perception, I’m pretty easy-going” (Female, age 56). Elsewhere another interviewee maintained that “Life just throws things at you and you just have to learn to… roll with it” (Female, age 53). Similarly, another said, “The way I looked at it is if you want something I still have to work” (Female, age 55). These participants’ agency stems from their adaptability to change, which can be due to their older age. If in the previous paragraph participants represented a higher self-reliance, in the quotes here there is a more flexible response. One possible explanation can be advanced age. It has been shown that despite the fact that people at older ages are usually exposed to more stressors related to their physical, mental, or social conditions, and as a result may seem more vulnerable, most manage to remain active and to age actively and successfully (Baltes and Freund 2003). Although there can be other reasons for such seemingly age-dependent responses such as cohort effects.

Very few of the participants held a negative attitude towards themselves, and the rare examples of such attitudes were usually expressed with a humorous tone to account for individual challenges. For example, one male participant said, “It’s like it’s kind of funny, my retirement plan is two Big Macs a week, I’m kind of hoping to drop dead some point within the next couple years because like I said I have no savings so… I’ll be so angry if I turn 60 [*laughing*]” (Male, age 51). Although few of the participants had this sense of humor, among all the interviews, only two participants started their life story negatively. As one explained, “It’d be pretty boring actually” (Male, age 54).

In some cases, participants expressed a belief in higher powers being in charge of their lives. One participant said she “leave[s] it up to the Provider and I do a lot of that too, a lot of self-prayer and a lot of motivational to keep my own” (Female, age 66). Her strong spirituality may stem from her heritage that was reflected in her opening lines: “I’m Metis raised…So, now I’m related to probably every reserve in [the province] […] you get the privileges of the status.” Her optimism is reflected in her sense of belonging: “Even though we’re going through what we are I know that we’ll get through because we have family; we do pull together the best we can.”

#### Communion

The experience of interpersonal connection (which McAdams and McLean [2013] describe as communion) was key to surviving economic cycles. As one participant explained, “It was always my mom. She was my biggest supporter…Even though we were poor, I didn’t feel that we were poor because we had family” (Female, age 63). Another participant said, “We didn’t have much but we were happy, we were together.[…] Ya. And we had love and ya we were safe so that’s really all that mattered” (Female, age 53). This emphasis on specific family members as a protective factor was consistent across the sample. While this fits the emphasis in resilience studies on the protective value of family (Masten 2014), it fits less neatly with understandings that economic pressure, including stressful experiences related to the imbalance between low income and financial demands (Berkowitz 1989), is one of the biggest stress factors in marriages and leads to problems for both individuals and the couple (Conger and Elder 1994).

#### Redemption & Meaning Making

In our sample, the ability to learn a positive lesson from a negative event (redemption) was common to many participants’ accounts of their lives. For some participants, periods of economic bust brought opportunities for new personal growth and better social cohesion. For example, one participant said, “The big positive is that I get to spend a lot of time with my family…I managed, I was able to stay home for the first year of my now-4-year-old’s life. And it was great, the bonding was just great” (Male, age 46). Although he sees his job loss and economic downturn as a negative state, he is also able to extract a positive outcome from such a situation. Another participant said, “Because my whole life as a child was not poverty but definitely not wealth, right? And I always knew and I didn’t have the stability of two parents […]” (Female, age 53). She used this negative experience to commit to something positive: “I always knew that whoever I would marry had better be a very loving, understanding and family first kind of man and so that’s who I married.” Economic volatility has encouraged robust personalities and a meta-narrative among residents of Maple Hill to explain their lives and the many hardships they have experienced as meaningful. Several participants characterized themselves as competent at handling hard times: “I am optimistic with life, […]you get hit in the head enough times, you kind of figure it out [chuckles]” (Female, age 65). Even poorly resourced single parents showed this same ability to make meaning out of hardship. As one woman said, “Because I’ve been a single parent for over 20 years, I’ve always been aware the need to prepare and not to be um, waiting for the next hand-out, because that hand-out may never come” (Female, age 55). Another explained that her life experience had taught her how to make meaning out of harsh and negative experiences and respect her own self, “You need to, whether you have children or not, you’re valuable, you’re important and whatever value you place on yourself will show on what supports you put around yourself. If you don’t value yourself, you won’t seek out help. I valued myself because I had two kids who I loved dearly” (Female, age 55). Finally, one participant simply said, “Well, I think you know just the concept of boom and bust and the stretch and the squeeze is that you do… you become more resilient or you die.[…] So, I think that it has certainly made me more resilient” (Female, age 49). Such descriptions suggest that adversity can trigger a re-evaluation of life priorities. Whether through a regenerated emotional bond with the family members, or through gaining new perspectives towards finances, or through leaning to appreciate little things in life, most of the participants demonstrated the ability to learn a lesson from difficulties which time and again supports their positive development.

#### Exploratory Narrative Processing

Under stress many of the participants’ life stories detailed a look back at past challenges while asserting that these challenges produced desirable outcomes. For example, one female participant, age 55, told us about how she chose to fight against an abusive husband, weak health, and very low financial resources: “As a result of that decision 9 years ago, I think I am stronger, I think I have a greater respect for what it means to appreciate life […] don’t give up, keep going, life can be better than you’ve ever seen” (Female, age 55). There is also evidence of meaning making through the highly agentic content of her narrative. She emphasized on multiple occasions how she chose to be a hero in her own story.

Another participant, a 70-year old man, explained why he didn’t leave the oil business as a sensible series of decisions over time, ones he recognized as risky but nevertheless were the best possible ones he could have made under the circumstances. As he said, “There were lots of times I wanted to get out of it but what do you do? You know? I was good at what I did I mean you know so….” While he may be retired, his narrative, like those of many others in Maple Hill, accounted for the boom and bust economy as a challenge from which to learn and grow.

#### Coherent Positive Resolution

Perhaps it was the participants’ collective emphasis on having a robust personality, or their penchant to narrate stories of personal redemption and growth, but far more of the stories told described coherent positive resolution of crises than despair and failure. As one woman explained, periods of economic downturn had stressed her marriage to a man who could not manage money so much that she had decided to separate from him. After a period of several years, though, she described the growth both she and her husband experienced and their eventual reunification: “I think we grew in that time like yes, we were apart but we grew to be better together” (Female, age 53). Another woman talked about periods of economic challenge but was careful to craft her account of that time as a period of personal transformation: “I guess [it is] humbling when you go to the store and you realize, okay I can only afford this and this and that […] but I… realize over the years that material things aren’t as important as having a roof over your head and a place to live with heat and running water and food” (Female, age 47). Even though economic cycles continued to challenge her and her family, she learned how to cope: “It seems like the cycles have been getting easier kind of. I don’t know if it’s easier or we’re just coping with it better to get through.”

#### Contamination

Though less common in the data, participants did occasionally narrate life stories that included positive experiences that turned bad or were ignored. To illustrate, an older woman recounted her regrets that she had not socialized more. “We kind of blew off a lot of our friends, and I don’t know why, […] now, I’m very lonely being a widow, ‘cause, I don’t have any friends [short laugh]” (Female, age 68). Countering this theme of contamination was a general belief in people’s abilities to perceive the positive even in contexts where their future was threatened. As one participant said, “You can make it work or it doesn’t matter what kind of job you have or whatever or what kind of place, you can be living in a palace and if your attitude’s not right” (Male, age 60). It is this pattern of attribution which seemed to buffer the impact of negative life events, even though it required an over-emphasis on agentic qualities of individuals to meet challenges that might be beyond their control.

## Discussion

How do adult residents of small towns that depend on oil and gas extraction or processing industries withstand economic boom and bust cycles? The collected narratives embodied many common elements related to the challenges and opportunities available in a single-industry town experiencing economic boom-bust cycles. As per the definition of resilience, challenges are a prerequisite to adaptive responses (Masten 2014). Summarizing patterns in our data, the most common adaptive response built on the theme of agency, a common factor found in resilience research (Masten and Motti-Stefanidi 2020; Rutter 2006). It is difficult to assert, however, whether the stress of living in Maple Hill created a “steeling effect” (Rutter 2006) that made the participants and their families and community more agentic, or whether our participants were a self-selected sample of individuals who chose to remain in an economically challenging environment, either because they had the skills to cope or were able to develop them. Regardless, life in towns like Maple Hill may require people to adopt the cognitive, social, and instrumental skills required to flexibly manage their lives under stress, whether that stress comes from economies that are booming or in recession. A number of personal and collective qualities of individuals in Maple Hill seem to be particularly adaptive. Participants changed jobs or daily habits and routines, including gendered roles, to survive changing circumstances. We observed only two participants who spoke more fatalistically, telling us they could do nothing but wait and see what comes next.

The second common theme was the role played by friends, family, and community supports. Belonging to a club or a group was repeatedly underscored as a source of resilience. Related to this theme and the construct of communion, participants talked about how much they care for their neighbors and the town’s motto, “Pull together.” Combined with narratives which featured redemptive sequences, people who cared more for others showed degrees of generative integration. Randall et al. (2015) have observed that generative integration means that our stories are connected to the world around us. Research on adult resilience supports the role of social connections and contextual support (Stewart and Yuen 2011). The value of social connections and contextual support fits with social–ecological understanding of resilience as a process that draws on more than personal resources (Ungar and Theron 2020; Masten and Motti-Stefanidi 2020). Put differently, this theme is a reminder that psychological resilience is intertwined with social and environmental resilience (Ungar and Theron 2020).

Third, residents showed high levels of meaning making in their narratives. The residents shared with us many of the lessons they have learned from weathering boom and bust cycles, and money management to reconsidering the value they place on family, church, and spirituality. Most notably, this pattern of meaning making started early in life and continued through each cycle of prosperity and ill-fortune, with the very fact that economic cycles occur with frequency being the catalyst for robust personalities and belief in a better future. As reported elsewhere (Masten and Motti-Stefanidi 2020), it is possible that this meaning making was enabled or sustained by the family and community systems that the residents were connected to.

Alongside these three life-story patterns, we observed some potential factors that may have shaped the participants’ resilience, either attenuating or accentuating their coping abilities. For instance, there was a sense that the culture of the industry and participants’ gender played a role. For instance, while financial difficulties affected the lives of both genders, multiple narratives referred to the pressure being stronger for men. One explanation would be the traditional ‘breadwinner’ mindset which is very dominant in the small community of Maple Hill. Similarly, Maple Hill’s culture of *big money* related to the oil and gas industry and its side effects was a repeated topic in almost all the interviews. Regardless of age, participants talked about a certain ‘attitude’ in Maple Hills, where those who earn ‘the big money’ seem to exclude those who are middle class from specific groups or gatherings. To better understand the potential influences of the effect of older age, the industry culture, and gender-related aspects, a secondary analysis of the data, and/or a follow-up study, would be useful.

## Limitations and Implications

Although this study fills a significant gap in existing knowledge about how resilience is experienced from the perspective of adult residents of an economically challenging environment, it also has some limitations. For example, in our sample, none of our participants had noticeable physical or cognitive limitations, such a sample may have presented us with different results or strategies of resilience. This would require additional research. Additionally, it would have been more precise to have interviewed the participants more than once and at various stages to have a point of comparison regarding developmental perspectives, which could also allow for their narratives to be more detailed and focused. This can be a future follow-up study.

Focusing on narratives, on the one hand, could become a tool for fostering resilience and helping the community to cope with the difficulties when it is used in the form of narrative care. Furthermore, the narratives also refer to the residents’ expectation and needs, such as implementing financial management in the education system, which can also be taken into consideration in future policies.

## Conclusion

Our study is an exemplar for a narrative understanding of how economic flux affects individual lives over time. This study based its analysis on narratives constructed by adult residents of a town dependent mostly on a single resource extraction industry and the volatile economic conditions that creates. While there was plenty of vulnerability to be found, our data also point to many promotive and protective factors that operate in this economically fragile community. The recurrence of agentic narratives coupled with a strong sense of communion seems to be the basis for a complex set of characteristics that make residents both fiercely optimistic and, at least in the short-term, resilient. Aside from their individual resources (agency, meaning making), the participants referred to the role of friends and family (communion), spiritually, and learning as the most valuable protective factors, and maintained that a more stable socio-economic context would eliminate some of the major difficulties they were experiencing in the first place.

However, we need to also point out that although much of the data points to the resilience of the population in Maple Hill, this positive orientation towards struggle and the future may inhibit communities like Maple Hill from diversifying their economies, even as the world shifts towards less dependence on oil and gas. Participants in this study may have been able to describe the intersecting forces at play when changing economic conditions transformed family relations, meaning making and identity, but that does not mean that they necessarily saw a need to fundamentally challenge the economic or social foundations of their community. We heard scant mention of the need for Maple Hill to adapt to permanent stagnation in the price of oil, or the need for a healthy pessimism regarding the prospects for future economic prosperity. Resilience was more aptly described as persistence (with governments colluding in the narrative of future prosperity) and resistance to negative thinking. Thus, the local narratives of resilience contrast with the need for thoughtful intervention by governments to direct the community towards a more diversified and economically resilient future. Aside from possible socio-economic interventions that the local government can program to mitigate the damage, as was also mentioned by some participants, there is a need for more fundamental trainings such as teaching children at school about budgeting and money management.

Finally, our results can help inform public policies that influence the mental health, social functioning, and economic sustainability of small towns that depend on a single resource extraction or processing industry. As changing social and economic environments put pressure on these towns to diversify their economies, it is useful to understand the interactions between individual psychological and social processes, economic factors, and natural ecologies if we are to both facilitate future economic development and prevent the large-scale displacement of populations affected by boom and bust economic cycles. The potential implications of how people adapt to financial shocks in life need further study in relation to public policy and welfare evaluation (Layard 2006; Loewenstein and Ubel 2008), specifically when we consider the fact that most of the risk factors are beyond the individual’s control and contextual.

## Electronic supplementary material

Below is the link to the electronic supplementary material.

Supplementary file1 (DOCX 15 kb)

## Data Availability

The interview guide is submitted as supplementary material. Any other material can be provided by the corresponding author upon inquiry.

## Footnotes

1 The name of the town has been changed to preserve the anonymity of the participants.

2 As the similarity of the terms may lead to some confusion, we will refer to the overall theories as narrative inquiry instead of narrative analysis. All the references to *narrative analysis* as a category of narrative inquiry will appear in italics.

3 This participant’s narrative is also included despite the relatively short residency due to the fact that her husband has been in the oil and gas industry for the last 20 years and therefore her narrative can still be representative of the experience.

## Code availability

ATLAS.ti 8 Software.
