# Supplementary material for: Resilience in Times of Economic Boom and Bust: A Narrative Study of a Rural Population Dependent upon the Oil and Gas Industry
Source: J Adult Dev. 2020 Oct 22;28(2):149–61. doi: 10.1007/s10804-020-09363-z (PMC8550031; doi:10.1007/s10804-020-09363-z)
Supplement: Supplementary file 1 — Supplementary file1 (DOCX 15 kb) [file 10804_2020_9363_MOESM1_ESM.docx]

**Appendix A.** **Interview Guide**

Introduction

We are interested in how the current situation of the oil and gas industry and past boom and bust cycles have influenced your life and the community of Maple Hill.

Introductory Questions Personal Info:

Can you start by telling us a bit about yourself?

Theme 1: Life Narratives

• I want to ask you to tell me about your life in a form of a story. Just imagine your life is a story, and in any way you like, just tell me about it.

Theme 2: Childhood

• We would like to know about your family life during your childhood. Where did you grow up? How would you describe your childhood at home?

• How was the financial situation of your parents? What did your parents work?

Theme 3: Private life

• How long have you lived in Maple Hill and what brought you here?

• How does the oil and gas industry affect your family life in relation to your partner, children and the family as a whole?

• What ways have you and your family found to handle the challenges of the boom and bust industry?

• Do you experience any positives for your family life during a bust?

• How does the oil and gas industry affect your social network in Maple Hill? Do you have the feeling that this industry influences who your friend is and who not?

Theme 4: Work life

• How is your job affected by the boom and bust cycles of the oil and gas industry?

Theme 5: Community life

• How would you describe the social life in Maple Hill? We heard that it is supposed to be very cliquey here. But is there still a feeling of being one community in Maple Hill?

• What are the positive effects or opportunities the oil and gas industry gives to Maple Hill?

• What challenges does the oil and gas industry place on Maple Hill?

Theme 6: Future

• What would you do if the oil price would return to the good old days tomorrow after experiencing this bust? Would you take the chance to leave for a more financially stable life?

If time left: some former questions:

• Drugs and crimes in Maple Hill?

• What additional resources would you like to see in the community for a better living?

• If you magically became the Mayor, what would you change to make Maple Hill a better place for you, your family and young people growing up in this community?

• Can you describe any positives in your and your family’s life that might be the result of the current situation of the oil and gas industry?

• Have you experienced any challenges during a boom on you and your family´s life?

• Do the stresses of the oil and gas production town affect women and men differently?

• What about older and younger people?

• What impact does someone’s wealth or the type of family they are from play on their level of

experience/ coping mechanism with regards to the boom and bust periods in (community name)?

• What about people from different cultures?

• What would Maple Hill be as a town if there was no more oil and gas industry? What’s

special about Maple Hill?

Closing/Concluding Questions

• Thinking back over the questions I have asked you and the stories you have shared is there

anything else you would like to tell me?

• Do you have any recommendations for these interview questions related to additional

questions we should be asking or questions we should be changing?
